# Detecting useful genetic markers and reconstructing the phylogeny of an important medicinal resource plant, *Artemisia selengensis*, based on chloroplast genomics

**DOI:** 10.1371/journal.pone.0211340

**Published:** 2019-02-04

**Authors:** Dong Meng, Zhou Xiaomei, Ku Wenzhen, Zhenggang Xu

**Affiliations:** 1 College of Materials and Chemical Engineering, Hunan City University, Yiyang, Hunan, China; 2 Hunan Research Center of Engineering Technology for Utilization of Environmental and Resources Plant, Central South University of Forestry and Technology, Changsha, China; National Cheng Kung University, TAIWAN

## Abstract

*Artemisia selengenesis* is not only a health food, but also a well-known traditional Chinese medicine. Only a fraction of the chloroplast (cp) genome data of *Artemisia* has been reported and chloroplast genomic materials have been widely used in genomic evolution studies, molecular marker development, and phylogenetic analysis of the genus *Artemisia*, which makes evolutionary studies, genetic improvement, and phylogenetic identification very difficult. In this study, the complete chloroplast genome of *A*. *selengensis* was compared with that of other species within *Artemisia* and phylogenetic analyses was conducted with other genera in the Asteraceae family. The results showed that *A*. *selengensis* is an AT-rich species and has a typical quadripartite structure that is 151,215 bp in length. Comparative genome analyses demonstrated that the available chloroplast genomes of species of *Artemisia* were well conserved in terms of genomic length, GC contents, and gene organization and order. However, some differences, which may indicate evolutionary events, were found, such as a re-inversion event within the *Artemisia* genus, an unequal duplicate phenomenon of the *ycf1* gene because of the expansion and contraction of the IR region, and the fast-evolving regions. Repeated sequences analysis showed that *Artemisia* chloroplast genomes presented a highly similar pattern of SSR or LDR distribution. A total of 257 SSRs and 42 LDRs were identified in the *A*. *selengensis* chloroplast genome. The phylogenetic analysis showed that *A*. *selengensis* was sister to *A*. *gmelinii*. The findings of this study will be valuable in further studies to understand the genetic diversity and evolutionary history of Asteraceae.

## Introduction

Asteraceae, the largest and the most diverse flowering plant family, currently has 32,913 accepted species in 1,911 genera and 13 subfamilies [1–3]. *Artemisia* L. (Asteraceae), as the largest genus in the Tribe Anthemideae, is widespread in mid- to high-latitudes and even dominates most cold and many warm deserts in the Northern Hemisphere. Numerous species of *Artemisia* are used as herbal medicines in many countries. For example, *A*. *annua* and *A*. *mexicana* produce antimalarial drugs [4–6], and artemisinin (from *A*. *annua*), first isolated and tested in the 1970s in China, is an active substance against malaria [7]. In particular, having good taste and rich nutrition, *A*. *selengenesis* has long been used as a health food source and is sometimes directly eaten. Some extracted substances, especially from the leaves and roots, have antitumor, antioxidant, and free radical scavenging activities, and the plant is also a well-known traditional medicine because of its potent effects [8, 9]. Therefore, considering the important medicinal values of *A*. *selengenesis* and the importance of *Artemisia* species as resource plants, comprehensive phylogenetic and genetic/genomic studies to increase our knowledge of this genus are important.

In angiosperms, the chloroplast with conserved quadripartite circular genomic structure [10] is a uniparentally inherited organelle. It originates from a cyanobacteria-like organism through an endosymbiosis event [11] and contains closely arrayed polycistronic transcribed gene clusters [12–14]. As a result, large-scale evolutionary events in related species, such as gene deletions or additions and gene order changes, are not common [15]. Therefore, cp genomes are widely used to determine evolutionary patterns [16], phylogenetic analysis [17], and comparative genomic analysis between angiosperm, gymnosperm, and fern families [18].

In the past, because of the number of species, diverse morphological types, ploidy, and complicated genetic relationships of *Artemisia*, the taxonomic relationships of the genus are controversial and based only on morphological traits, such as the capitula type and floret fertility[19, 20]. As a result, considering the conserved structural and relatively compact gene density, chloroplast genomic materials are widely used in genomic evolution studies, molecular marker development, and phylogenetic analysis of the genus *Artemisia*. Many researchers have used single gene data (*matK*, *ndhF*, *rps11*), IGS data (*psbA*_*trnH*, *trnS*_*trnC*, *trnS*_*trnfM*, *trnL*_*trnF*), and shared protein-coding gene data of *Artemisia* to perform phylogenetic analysis [19–27]. However, the cp genomic data of *Artemisia* are still quite limited and data for only a few species have been reported.

Therefore, we sequenced and annotated the complete cp genome of *A*. *selengensis* and compared it with other species within *Artemisia* and other genera (*Chrysanthemum*, *Soliva*, *Diplostephium*, *Cynara*) within the Asteraceae family. Our study aimed to detect useful genetic markers and genetic materials, and to reconstruct its phylogeny. This study will be useful in further studies in that it will illuminate the genetic diversity and evolutionary history of Asteraceae.

## Materials and methods

### Ethics statement

The plant sampling was collected in areas that were not privately owned or protected in any way and no specific permits were required for this study.

### Plant material and high throughput sequencing

The sample was collected from the Dongting Lake region (28°48′46.06″N, 112°21′10.19″E). Firstly, we collected mature leaves of *A*. *selengensis* and put them in a container with liquid nitrogen. Then, leaves were stored at -80°C until sequencing. The extraction of total cp DNA was conducted according to the method of Zhang [28].

### Chloroplast genome assembly and annotation

The cp DNA of *A*. *selengensis* was fragmented using Covaris M220 (Covaris, USA). The whole-genome sequencing and the PE library construction was conducted according to the method of Zhang [29]. Approximately 2G of raw data were obtained through next generation sequencing with paired-end 125 bp read length. After filtering using Trimmomatic v 0.32, clean data were obtained for subsequent analysis [30].

The quality of the sequencing data of the samples was visually evaluated using the software Fastqc v 0.10.0 and low-quality reads were filtered using quality control [31]. Then, we used SOAP denovo2 to assemble all good-quality paired reads to contigs [32]. Assembled contigs were joined into multiple scaffolding using SSPACE [33] to obtain the whole-genome sequence. In this process, different K-mers were selected firstly for assembly, the best k-mer was obtained to adjust the other parameters (-d -u -R -F, etc.), and then the preliminary assembly results were obtained again. Finally, GapCloser [32] software was used for optimization and gap filling to obtain the final assembly results. We filtered out fragments below 500 bp for evaluation, statistical analysis, and subsequent gene prediction.

The predicted annotation of the complete cp genome was performed by using the programs CpGAVAS and DOGMA [34] with default values. Then, the annotation results were stored in GFF3 format and checked manually, and codon positions were adjusted using Apollo [35]. OGDraw v1.2 [36, 37] was used to visualize the gene features of the *A*. *selengensis* genome. The other more details about material collection, sequencing, annotation can be obtained from the announcement[38]. Furthermore, codon usage and the relative synonymous codon usage (RSCU) of the *A*. *selengensis* cp genome were confirmed using DAMBE6 [39] based on the protein-coding sequences.

### Comparative analysis

Over the course of evolution, genomes can undergo many small and large-scale changes. To find large-scale evolutionary events in *A*. *selengensis*, we analyzed the genome rearrangement and the contraction/expansion of the IRs regions by comparing them with that of 8 related species in the Asteraceae family, as follows: *A*. *capillaris* (KU736963) [25], *A*. *frigida* (JX293720) [23], *A*. *gmelinii* (KU736962) [25], *A*. *montana* (KF887960), *Chrysanthemum boreale* (MG913594) [40], *S*. *sessilis* (KX063863) [41], *D*. *glutinosum* (KX063897) [41], and *C*. *humilis* (KP299292) [42]. The genome rearrangement analyses of nine Asteraceae species relative to *C*. *humilis* was performed in Mauve Alignment [43]. The contraction/expansion of the IRs regions of the nine Asteraceae species relative to *A*. *selengensis* was visualized using Microsoft Visio 2016.

To obtain comprehensive knowledge of the genomic variation, pairwise distances of intergenic spacers (IGSs), and introns, protein-coding sequences of the nine Asteraceae species relative to *A*. *selengensis* were calculated. First, we extracted a total of 83 IGSs with at least 100 bp, and 17 introns shared by these species, and performed sequence alignment using MAFFT v7.380 [44] under the FFT-NS-2 setting. At the same time, 80 protein-coding sequences were extracted and aligned in MEGA7 [45] with the ClustalW (Codons) program. Then, pairwise distances of IGSs and introns were determined by using MEGA7 [45] with Kimura’s two parameter (K2P) model [46]. Additionally, sequence divergence of homologous protein-coding genes was estimated according to Keller’s method [47] using the synonymous (Ks) and non-synonymous (Ka) nucleotide substitution rates with the yn00 program [48] from the PAML package [49]. Finally, a two independent samples t-test was performed to evaluate the significance of the Ka/Ks ratio within and outside of the genus *Artemisia*.

### Repeated sequences analysis

We detected the type and number of repeated sequences across nine Asteraceae species (*A*. *selengensis*, *A*. *capillaris*, *A*. *frigida*, *A*. *gmelinii*, *A*. *montana*, *C*. *boreale*, *S*. *sessilis*, *D*. *glutinosum*, *C*. *humilis*) to find useful genetic/genomic makers. These repeated sequences were divided into two categories: (i) simple sequence repeats (SSRs or microsatellites) with 1–6 bp long repeat motifs, (ii) longer dispersed repeats (LDRs) with at least 30 bp long repeat motifs. We used MISA Perl Script [50] that was written by a Perl program to determine SSRs in the *A*. *selengensis* cp genome. The minimum number of repeats was set to 8, 4, 4, 3, 3, 3 for mono-, di-, tri-, tetra-, penta-, and hexa-nucleotide SSRs, respectively. Then, LDRs, including tandem (T), forward (F), palindrome (P), reverse (R), and complement (C) repeats, were identified. Tandem Repeats Finder version 4.09 [51] with default settings was used to detect tandem repeats. These repeats with n ≥30 bp and a sequence identity ≥ 90% were selected. REPuter [52] was used to visualize forward, palindrome, reverse, and complement sequences with the parameter settings of 3 for Hamming distance and 30 bp for minimum repeat size.

### Phylogenetic analysis

Five datasets, including the complete cp genome, LSC, IR, and SSC DNA sequences, and 72 shared protein sequences of 28 published Asteraceae species and *A*. *selengensis*, were used to accomplish the phylogenetic analysis. The neighbor-joining (NJ) method was used to determine the phylogenetic relationships. The probability bootstrap analysis of each branch was calculated with 1000 replications. The online software Interactive Tree of Life (iTOL) was used to construct a phylogeny tree. The species were *Artemisia annua* (MF623173), *Artemisia argyi* (KM386991), *A*. *capillaris*, *A*. *frigida*, *Artemisia fukudo* (KU360270), *A*. *gmelinii*, *A*. *montana*, *C*. *boreale*, *Chrysanthemum indicum* (JN867589), *Chrysanthemum x morifolium* (JQ362483), *S*. *sessilis*, *Leontopodium leiolepis* (KM267636), *Anaphalis sinica* (KX148081), *Aster spathulifolius* (KF279514), *D*. *glutinosum*, *Diplostephium romeroi* (KX063911), *Heterothalamus alienus* (KX063869), *Oritrophium peruvianum* (KX063861), *Conyza bonariensis* (MF276802), *Hinterhubera ericoides* (KX063910), *Laestadia muscicola* (KX063873), *Floscaldasia hypsophila* (KX063916), *Archibaccharis asperifolia* (KX063859), *Lagenophora cuchumatanica* (KX063879), *Guizotia abyssinica* (EU549769), *Mikania micrantha* (KX154571), *C*. *cornigera*, and *C humilis*. *C*. *cornigera* and *C*. *humilis* were selected as the out group.

BLAST 2.8.1 [53] was used to align and perform NJ analyses of the complete cp genome, LSC, IR, and SSC DNA sequences, together with MEGA 7.0 [54] for 72 shared protein sequences alignment and NJ analyses. The results were stored as a Newick tree file for constructing a phylogeny tree.

## Results

### Features of complete chloroplast genome

The *A*. *selengensis* cp genome with GenBank accession number: MH042532 was announced by our research group[38]. The complete cp genome of *A*. *selengensis* had a typical quadripartite structure and was 151,215 bp in length (Table 1). The GC content of the whole genome, LSC, SSC, and IR regions were 37.46%, 35.55%, 30.81%, and 43.09%, respectively. The higher GC content of the IR regions was probably caused by the presence of all four ribosomal RNA genes duplicated in these regions [55] (Table 1). Furthermore, AT content of the 1st, 2nd, and 3rd positions of the codons were 54.1%, 61.9%, and 70.2%, respectively (Table 1).

**Table 1:** Base compositions in the *A*. *selengensis* chloroplast genome.

Location	T/U (%)	C (%)	A (%)	G (%)	Length (bp)
Genome	31.28	18.67	31.26	18.79	151215
tRNA genes	22.66	26.73	24.59	26.02	2798
rRNA gens	22.46	27.54	22.46	27.54	9048
Introns region	32.31	18.86	30.75	18.07	17240
Protein-coding genes	31.54	17.75	30.53	20.19	77778
Intergenic region	33.34	16.07	34.23	16.36	44274
1st positon	23.50	19.08	30.58	26.84	25926
2nd positon	32.70	20.39	29.21	17.70	25926
1st+2nd positon	28.10	19.73	29.90	22.27	51852
3rd positon	38.42	13.78	31.78	16.02	25926

**Fig 1:**
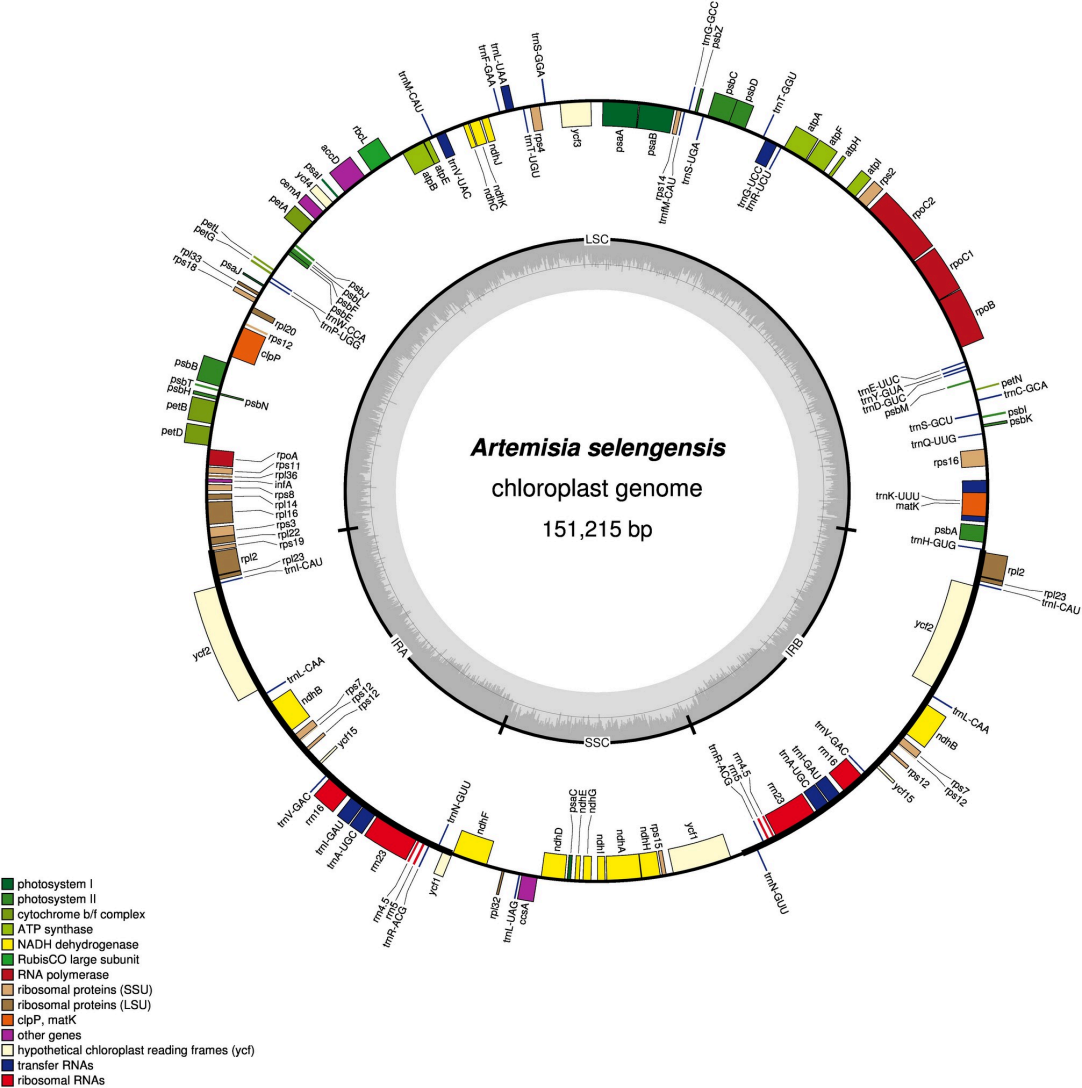
Gene map of the complete chloroplast genome of *A*. *selengensis*. Genes lying inside of the circle are transcribed clockwise, and those outside are transcribed counterclockwise. Different color of blocks represent different functional groups. The darker gray color of the inner circle corresponds to the GC content, and the lighter gray color corresponds to the AT content.

A total of 114 unique genes, including 80 protein-coding, 30 tRNA, and four rRNA genes, were found (Fig 1, Table 2). Among these genes, 19 genes (*atpF*, *clpP*, *ndhA*, *ndhB*×2, *petB*, *petD*, *rpl2*×2, *rpl16*, *rps16*, *rpoC1*, *trnA-UGC*×2, *trnG-UCC*, *trnK-UUU*, *trnI-GAU*×2, *trnL-UAA*, *trnV-UAC*, *ycf3*) contained a single intron, whereas two genes, *ycf3* and *clpP*, contained two introns (Table 3).

**Table 2:** Genes predicted in the chloroplast genome of *A*. *Selengensis*.

Category	Group of genes	Name of genes
Self-replication	Large subunit of ribosomal proteins	*rpl2* ^*a*^, *rpl14*, *rpl16*, *rpl20*, *rpl22*,*rpl23* ^*a*^, *rpl32*, *rpl33*, *rpl36*
	Small subunit of ribosomal proteins	*rps2*, *rps3*, *rps4*, *rps7* ^*a*^, *rps8*, *rps11*, *rps12* ^*a*^ ^b^, *rps14*, *rps15*, *rps16*, *rps18*, *rps19*
	DNA dependent RNA polymerase	*rpoA*, *rpoB*, *rpoC1*, *rpoC2*
	rRNA genes	*rrn16S* ^*a*^, *rrn4*.*5S* ^*a*^, *rrn5S* ^*a*^, *rrn23* ^a^
	tRNA genes	*trnA-TGC* ^*a*^, *trnC-GCA*, *trnD-GTC*, *trnE-TTC*, *trnF-GAA*, *trnfM-CAT*, *trnG-GCC*, *trnG-TCC*, *trnH-GTG*, *trnI-CAT* ^*a*^, *trnI-GAT* ^*a*^, *trnK-TTT*, *trnL-CAA* ^*a*^, *trnL-TAA*, *trnL-TAG*, *trnM-CAT*, *trnN-GTT* ^*a*^, *trnP-TGG*, *trnQ-TTG*, *trnR-ACG* ^*a*^, *trnR-TCT*, *trnS-GCT*, *trnS-GGA*, *trnS-TGA*, *trnT-GGT*, *trnT-TGT*, *trnV-GAC* ^*a*^, *trnV-TAC*, *trnW-CCA*, *trnY-GTA*
Photosynthesis	Photosystem I	*psaA*, *psaB*, *psaC*, *psaI*, *psaJ*
	Photosystem II	*psbA*, *psbB*, *psbC*, *psbD*, *psbE*, *psbF*, *psbH*, *psbI*, *psbJ*, *psbK*, *psbL*, *psbM*, *psbN*, *psbT*, *psbZ*
	NADH dehydrogenase	*ndhA*, *ndhB* ^*a*^, *ndhC*, *ndhD*, *ndhE*, *ndhF*, *ndhG*, *ndhH*, *ndhI*, *ndhJ*, *ndhK*
	Cytochrome b6/f complex	*petA*, *petB*, *petD*, *petG*, *petL*, *petN*
	ATP synthase	*atpA*, *atpB*, *atpE*, *atpF*, *atpH*, *atpI*
	Rubisco	*rbcL*
Other genes	Translational initiation factor	*infA*
	Maturase	*matK*
	Protease	*clpP*
	Envelop membrane protein	*cemA*
	Subunit Acetyl-CoA-Carboxylase	*accD*
	C-type cytochrome synthesis gene	*ccsA*
Genes of unkown function	Conserved Open reading frames	*ycf1*^*a*^, *ycf2* ^*a*^, *ycf3*, *ycf4*, *ycf15* ^*a*^

^a^ Duplicated gene

^b^ Trans-splicing gene.

**Table 3:** Length of introns and exons of the split genes in the *A*. *Selengensis* complete chloroplast genome.

Gene Name	Gene Location			Length (bp)				
	Strand	Start	End	Exon I	Intro I	Exon II	Intro II	Exon III
*rps16*	-	5190	6275	40	861	185		
*rpoC1*	+	15912	18705	432	721	1641		
*atpF*	+	26621	27874	145	699	410		
*ycf3*	-	41826	43775	126	703	228	740	153
*clpP*	-	68800	70794	68	798	292	609	228
*petB*	+	73721	75113	6	745	642		
*petD*	+	75302	76459	8	675	475		
*rpl16*	-	79921	81347	9	1019	399		
*rpl2*	-	83042	84530	393	661	435		
*ndhB*	-	93079	95281	777	670	756		
*ndhA*	-	117648	119820	553	1081	539		
*ndhB*	+	138855	141057	777	670	756		
*rpl2*	+	149606	151094	393	661	435		
*trnK-UUU*	-	1722	4340	37	2547	35		
*trnG-UCC*	-	29908	30705	23	728	47		
*trnL-UAA*	+	46606	47116	37	424	50		
*trnV-UAC*	-	51073	51719	38	572	37		
*trnI-GAU*	+	100805	101657	43	775	35		
*trnA-UGC*	+	101722	102606	38	812	35		
*trnA-UGC*	-	131530	132414	38	812	35		
*trnI-GAU*	-	132479	133331	43	775	35		

A total of 25,926 codons were translated into 88 protein-coding sequences by 30 unique tRNA genes (Tables 1 and 4). By analyzing codon usage and the relative synonymous codon usage (RSCU) of protein-coding sequences of the *A*. *selengensis* cp genome, we found that AUU and UGC accounted for the highest and lowest codon usage, respectively. Furthermore, non-preferred synonymous codons (RSCU < 1) with 32 codons is more than preferred synonymous codons (RSCU > 1) with 28 codons. The start codon AUG and UGG were non-bias codons (RSCU = 1). We also found that all preferred synonymous codons ended with A/T nucleotides and 93.75% non-preferred synonymous codons ended with G/C (Table 4).

**Table 4:** The codon-anticodon recognition pattern and codon usage for *A*.*Selengensis* chloroplast genomeAnimo acid.

Animo acid	Codon	No.	RSCU	tRNA	Animo acid	Codon	No.	RSCU	tRNA
Ala	GCU	365	1.565	trnA-UGC	Pro	CCA	409	1.504	trnP-UGG
Ala	GCG	132	0.566		Pro	CCC	236	0.868	
Ala	GCC	210	0.9		Pro	CCU	306	1.125	
Ala	GCA	226	0.969		Pro	CCG	137	0.504	
Cys	UGU	305	1.063	trnC-GCA	Gln	CAA	630	1.491	trnQ-UUG
Cys	UGC	269	0.937		Gln	CAG	215	0.509	
Asp	GAU	642	1.566	trnD-GUC	Arg	AGA	518	1.265	trnR-ACG
Asp	GAC	178	0.434		Arg	AGG	301	0.735	trnR-UCU
Glu	GAG	263	0.517	trnE-UUC	Arg	CGA	240	1.299	
Glu	GAA	755	1.483		Arg	CGC	125	0.677	
Phe	UUU	984	1.15	trnF-GAA	Arg	CGG	140	0.758	
Phe	UUC	728	0.85		Arg	CGU	234	1.267	
Gly	GGU	411	1.185	trnG-GCC	Ser	AGC	365	0.892	trnS-GCU
Gly	GGG	256	0.738	trnG-UCC	Ser	AGU	453	1.108	trnS-GGA
Gly	GGC	203	0.585		Ser	UCA	182	0.491	trnS-UGA
Gly	GGA	517	1.491		Ser	UCC	502	1.354	
His	CAC	149	0.423	trnH-GUG	Ser	UCG	266	0.717	
His	CAU	555	1.577		Ser	UCU	533	1.438	
Ile	AUU	1031	1.294	trnI-CAU	Thr	ACC	413	1.151	trnT-GGU
Ile	AUA	715	0.897	trnI-GAU	Thr	ACA	301	0.839	trnT-UGU
Ile	AUC	644	0.808		Thr	ACG	238	0.663	
Lys	AAA	988	1.332	trnK-UUU	Thr	ACU	483	1.346	
Lys	AAG	495	0.668		Val	GUU	403	1.387	trnV-GAC
Leu	CUA	184	0.648	trnL-CAA	Val	GUG	186	0.64	trnV-UAC
Leu	CUC	261	0.92	trnL-UAA	Val	GUC	206	0.709	
Leu	CUG	205	0.722	trnL-UAG	Val	GUA	367	1.263	
Leu	CUU	485	1.709		Trp	UGG	376	1	trnW-CCA
Leu	UUA	433	0.785		Tyr	UAC	339	0.61	trnY-GUA
Leu	UUG	670	1.215		Tyr	UAU	773	1.39	
Met	AUG	528	1	trnM-CAU	*	UGA	237	0.763	
Asn	AAC	383	0.54	trnN-GUU	*	UAG	202	0.65	
Asn	AAU	1035	1.46		*	UAA	493	1.587	

The asterisk (*) means stop codon.

### Comparative chloroplast genomic analysis

#### Genome features comparation of nine Asteraceae species

We compared *A*. *selengensis* with its related species, including four species from *Artemisia* and four species from other genera: *Chrysanthemum*, *Soliva*, *Diplostephium*, and *Cynara* (Table 5). Among them, the length of the cp genomes of the nine species ranged from 150,784 (S. *sessilis*) bp to 152,585 bp (*C*. *humilis*). The genomic length within the *Artemisia* genus was similar, ranging from 151,056 bp (*A*. *capillaris*) to 151,318 bp (*A*. *gmelinii*) with only a 255 bp difference. The LSC region accounted for 54.77%–54.89% of the whole genome, whereas the SSC and IRs regions accounted for 12.12%–12.17% and 16.50%–16.53%, respectively. In terms of gene organization, *Artemisia* species appeared to be well conserved with 21 genes containing introns and 114 unique genes, including 80 protein-coding genes, 30 tRNA genes, and four rRNA genes.

**Table 5:** Characteristics of nine Asteraceae species.

Species	*A*. *selengensis*	*A*. *capillaris*	*A*. *frigida*	*A*. *gmelinii*	*A*. *montana*	*C*. *boreale*	*S*. *sessilis*	*D*. *glutinosum*	*C*. *humilis*
Length (bp)/GC content (%)	151215/37.46	151056/37.46	151076/37.48	151318/37.42	151130/37.48	151012/37.47	150784/37.46	152229/37.33	152585/37.70
Size (bp)/GC content (%) of LSC	82920/35.55	82821/35.56	82740/35.58	83061/35.49	82873/35.57	82817/35.56	82958/35.51	83954/35.32	83622/35.82
Size (bp)/GC content (%) of SSC	18367/30.81	18309/30.72	18392/30.83	18335/30.83	18339/30.87	18281/30.85	18338/31.12	18233/31.11	18651/31.51
Size (bp)/GC content (%) of IR	24964/43.09	24963/43.08	24972/43.06	24961/43.06	24959/43.08	24957/43.08	24744/43.10	25021/42.97	25156/43.13
Size (bp)/GC content (%) of CDS	77928/37.84	79197/37.71	79182/37.77	79167/37.76	78912/37.77	76983/38.02	78372/37.75	78771/37.88	80257/38.03
Size (bp)/GC content (%) of introns	17240/36.94	17244/36.92	17259/36.93	17303/36.85	17308/36.88	15524/37.74	16197/37.41	16479/37.18	16200/37.28
Size (bp)/GC content (%) of rRNA	9048/55.08	9048/55.08	9048/55.08	9048/55.08	9048/55.08	9048/55.08	9047/55.18	9047/55.18	9046/55.23
Size (bp)/GC content (%) of tRNA	2798/52.75	2798/52.72	2806/52.67	2798/52.75	2806/52.71	2723/52.63	2692/52.45	2694/52.86	2726/52.93
Size (bp)/GC content (%) of IGSs	44274/32.43	42872/32.48	42854/32.44	43075/32.34	43129/32.50	46807/32.20	44549/32.49	45311/31.94	44446/32.77
No. of different genes	114	114	114	114	114	113	111	111	114
No. of different protein-coding genes *	80	80	80	80	80	80	79	79	81
No. of different rRNA genes	4	4	4	4	4	4	4	4	4
No. of different tRNA genes	30	30	30	30	30	29	28	28	29
No. of different duplicated genes by IR	18	19	20	19	19	18	19	19	21
No. of genes with introns **	21	21	21	21	21	19	20	20	20

* The presence of pseudogenes in the complete genome of *A*. *frigida*, *A*. *montana*, *S*. *sessilis*, *D*. *glutinosum* (*ycf1*, *rps19*), *and C*. *humilis* (*ycf1*, *ycf68*, *rps19*).

** Introns losses: one intron missing in *rpl16* (*C*. *boreale*, *S*. *sessilis* and *D*. *glutinosum*).

#### Large-scale evolutionary events in the chloroplast genome of *A*. *selengensis*

**Fig 2:**
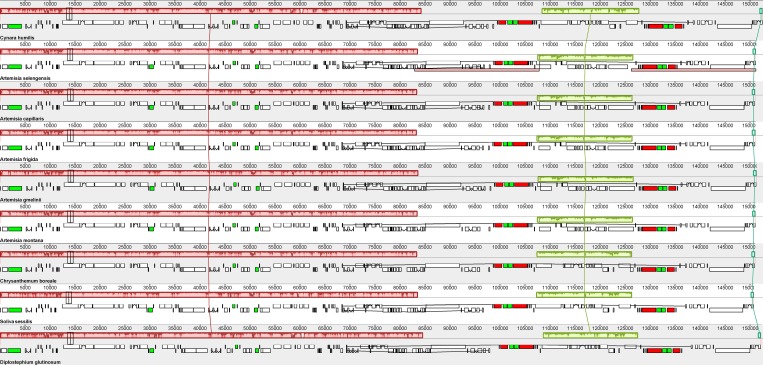
Genomic rearrangement of nine Asteraceae species relative to *C*. *humilis*. Locally collinear blocks (LCBs) are colored to indicate syntenic regions. Homologous sequences are connected with the same color strand. Histograms of each LCBs corresponds to sequence similarity. Blocks below the center line indicate regions that align in the reverse complement (inverse) orientation. The small boxes below the LCBs of each chloroplast genome are represented as genes.

Additionally, the genomic rearrangement of nine Asteraceae species relative to *C*. *humilis* showed that the SSC region of five species within the *Artemisia* genus had no rearrangement but was inverted in comparison with other genera. All species in our study were highly syntenic and similar in their LSC and IRs regions (Fig 2).

**Fig 3:**
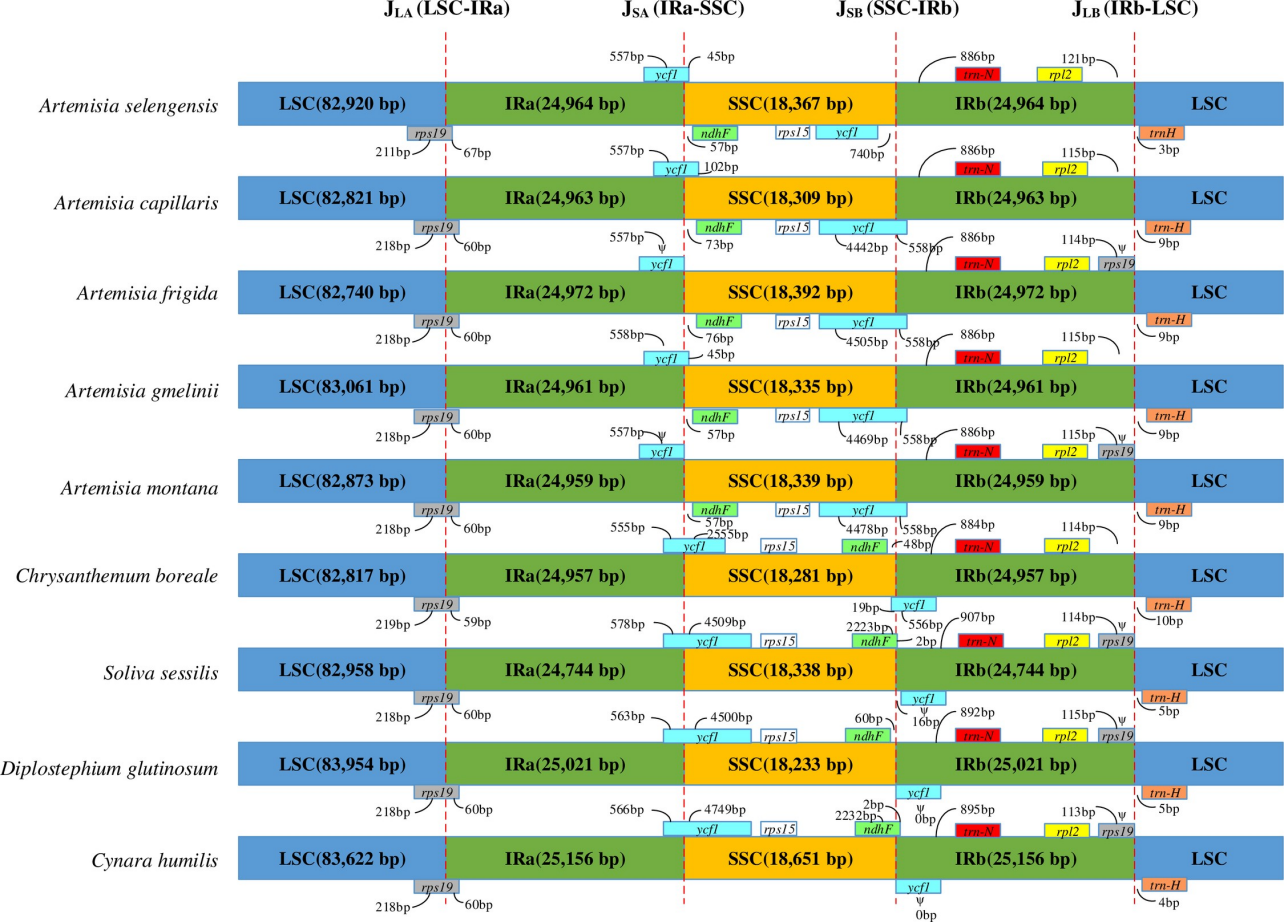
The expansion and contraction of the inverted repeats (IRs) of nine Asteraceae species relative to *A*. *selengensis*. The small boxes of each chloroplast genome are represented as genes. Genes above the larger box correspond to their transcriptions in forward direction and genes below the larger box represent their transcriptions in reverse direction.

The expansion and contraction of the IR region was the most common evolutionary event in the evolution of the genome, and they are hypothesized to explain size differences between cp genomes [24]. Therefore, we compared the IR/SSC and IR/LSC boundaries of the nine species relative to *A*. *selengensis* (Fig 3). The LSC/IRa border generally was positioned at the *rps19* gene with 211–218 bp in LSC, 60–67 bp in IRa. Normally, *rpl2* and *trn-H* are positioned at the IRb/SSC boundary, but we also found a pseudogene *rps19* at the IRb/SSC boundary of *A*. *frigida*, *A*. *montana*, *S*. *sessilis*, *D*. *glutinosum*, and *C*. *humilis*. The IRa/SSC and SSC/IRb borders of intro-generic species and inter-generic species were different because of different gene order in SSC. In our study, the *ycf1* gene had a duplicate phenomenon in the cp genome, but the length of these two genes were different. The shorter one set as *ycf1*_1 ranged from 557 to 660 bp, and the longer one set as *ycf1*_2 ranged from 3,111 to 5,085 bp. In intro-generic species, *ycf1*_1 and *ndhF* were located at the IRa/SSC border, whereas *rps15* and *ycf1*_2 were at the SSC/IRb border, which was opposite in inter-generic species. The pseudogene *ycf1*_1, ranging from 557 to 558 bp, in *A*. *frigida*, *A*. *montana*, *S*. *sessilis*, *D*. *glutinosum*, and *C*. *humilis* was expressed in four species, ranging from 576 to 660 bp. It is hypothesized that the *ycf1* gene plays an important role in genome evolution. We also found that the *ycf1* gene overlapped with the *ndhF* gene at the IRa/SSC boundary in *A*. *capillaris* and the SSC-IRb boundary in *C*. *humilis*.

#### Sequence divergence between intro-generic species and inter-generic species

**Fig 4:**
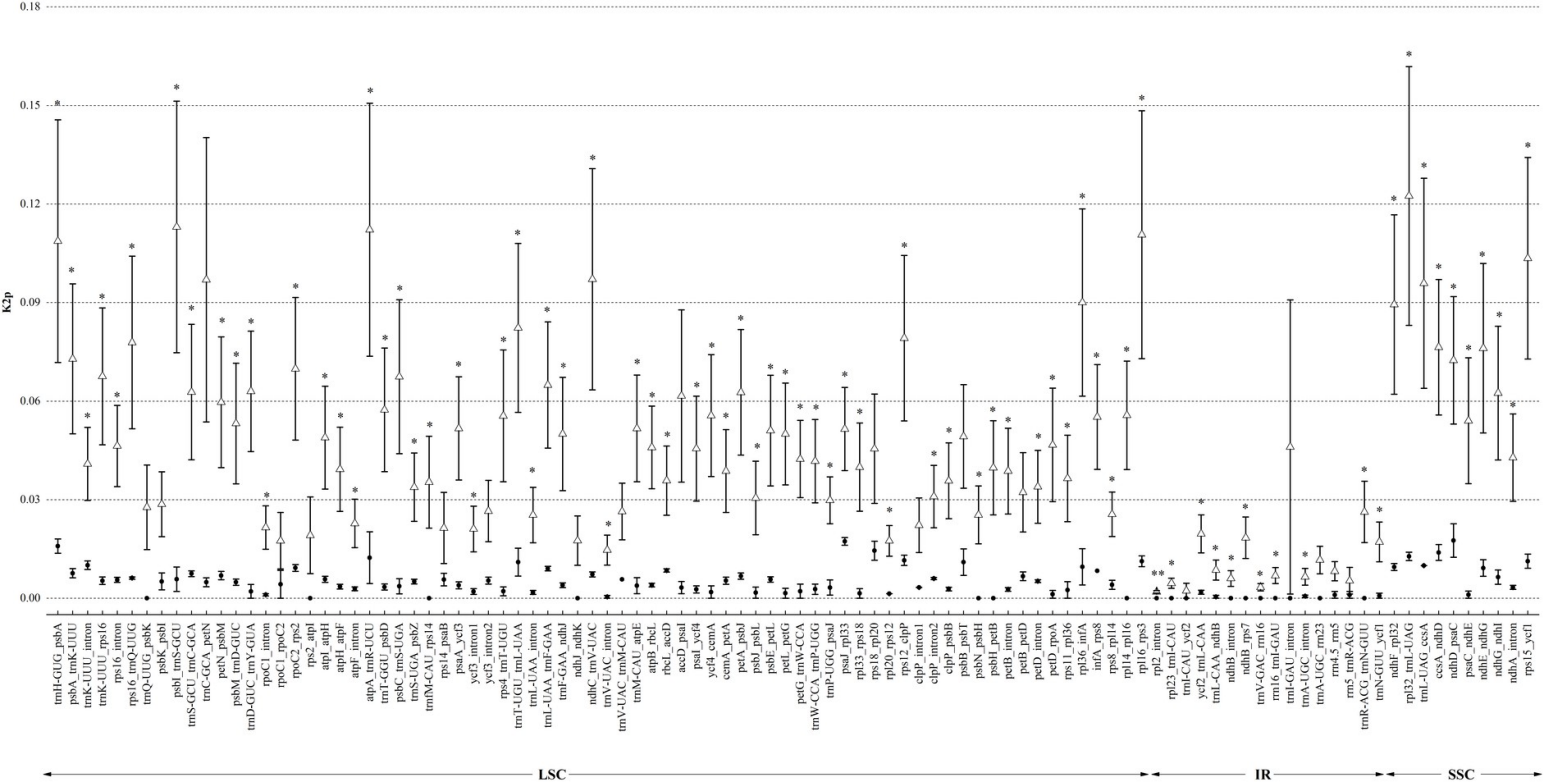
Kimura’s two parameter (K2p) values of introns and intergenic spacers (IGSs) between intro-generic species (within *Artemisia*: *A*. *selengensis*, *A*. *capillaris*, *A*. *frigida*, *A*. *gmelinii*, *A*. *montana*) and inter-generic species (other genus of Asteraceae: *A*. *selengensis*, *C*. *boreale*, *S*. *sessilis*, *D*. *glutinosum*, *C*. *humilis*). Black circles represent the mean K2p values of intro-generic species, and blank triangles indicate the mean K2p values of inter-generic species. Bars are mean values (±SE, n = 5). Symbols indicate levels of statistical significance between intro-generic species and inter-generic species: no symbol P > 0.05; *P = 0.01–0.05; **P < 0.01. X-axis denotes the homologous regions arranged by position.

**Fig 5:**
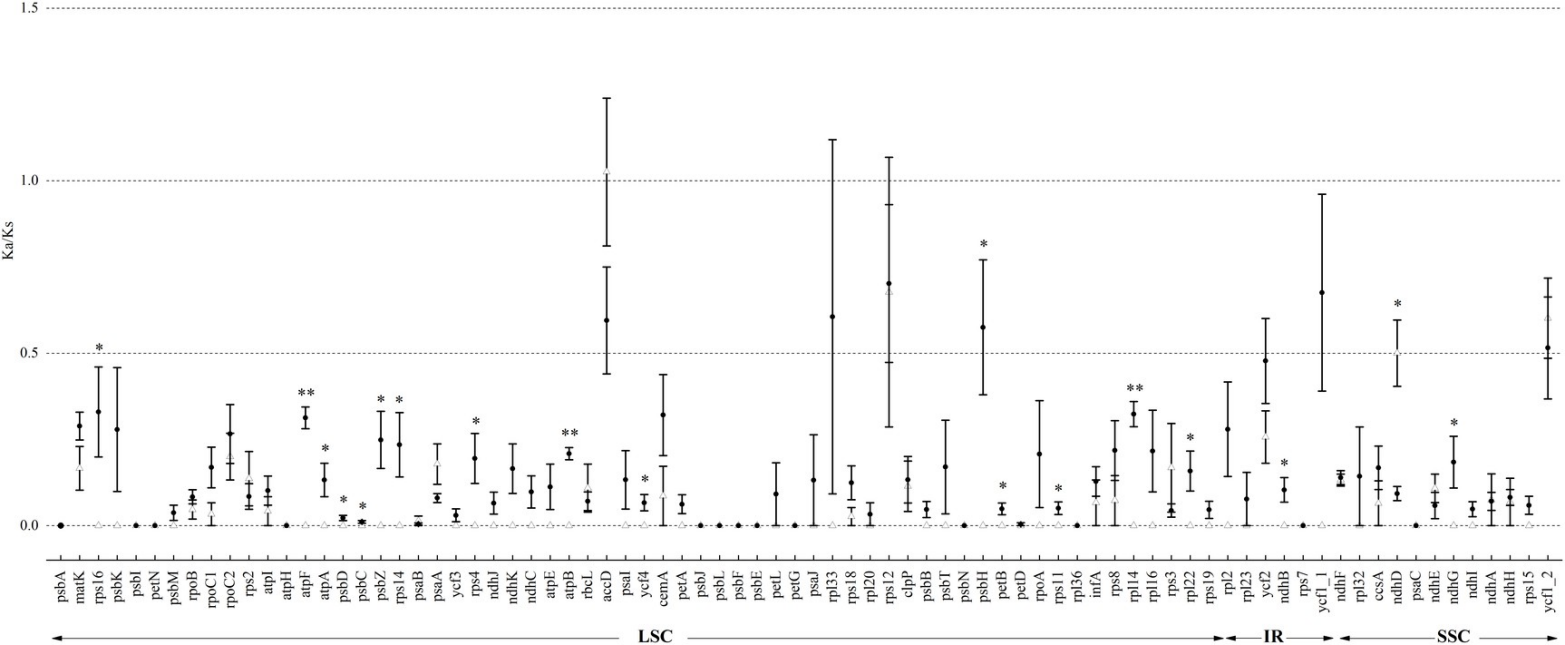
Ka/Ks ratio of protein-coding genes between intro-generic species (within *Artemisia*: *A*. *selengensis*, *A*. *capillaris*, *A*. *frigida*, *A*. *gmelinii*, *A*. *montana*) and inter-generic species (other genus of Asteraceae: *A*. *selengensis*, *C*. *boreale*, *S*. *sessilis*, *D*. *glutinosum*, *C*. *humilis*). Black circles represent the mean Ka/Ks values of intro-generic species, and blank triangles indicate the mean Ka/Ks values of inter-generic species. Bars are mean values (±SE, n = 5). Symbols indicate levels of statistical significance between intro-generic species and inter-generic species: no symbol P > 0.05; *P = 0.01–0.05; **P < 0.01. X-axis denotes the homologous genes arranged by position.

To obtain a comprehensive knowledge on the variation in the protein-coding genes, introns, and intergenic spacers in the cp genome, we compared the K2p values of the intergenic spacers and introns and the Ka, Ks, and Ka/Ks ratio of the protein-coding genes of the nine Asteraceae species (Figs 4 and 5; S1, S2, S3 and S4 Tables). These species were divided into intro-generic species (within *Artemisia*: *A*. *selengensis*, *A*. *capillaris*, *A*. *frigida*, *A*. *gmelinii*, and *A*. *montana*) and inter-generic species (other genera of Asteraceae: *A*. *selengensis*, *C*. *boreale*, *S*. *sessilis*, *D*. *glutinosum*, and *C*. *humilis*). As excepted, the IR region was much more conserved than the LSC and SSC regions because of lower K2p values. The sequences differences between species weresignificantly higher than those of the species within the genus (P < 0.05). In intro-generic species, *ndhD*_*psaC* (116 bp), *psaJ*_*rpl33* (439 bp), *trnH-GUG*_*psbA* (382 bp), *rps18*_*rpl20* (264 bp), *ccsA*_*ndhD* (200 bp), and *rpl32*_*trnL-UAG* (880 bp) presented higher K2p values. The most variable intron in *Artemisia* was *trnK-UUU* and the second intron of *clpP*. The most divergence intergenic sequences between *A*. *selengensis* and species of other genera were *rpl32*_*trnL-UAG*, *psbI*_*trnS-GCU*, *atpA*_*trnR-UCU*, *rpl16*_*rps3*, and *trnH-GUG*_*psbA*, whereas the most variable intron was *rps16* (Fig 4).

A comparison among the protein-coding genes showed that the mean Ka among the five *Artemisia* species ranged from 0 (contained 45 genes) to *psbH* (0.0119) and the mean Ks ranged from 0 (contained 29 genes) to 0.0316 (*infA*). However, the mean Ka among *A*. *selengensis* and other genera ranged from 0 (*atpH*, *petG*, *petN*, *psaC*, *psbA*, *psbE*, *psbF*, *psbI*, *psbJ*, *psbL*, *rpl36*) to 0.0533 (*ycf1*_2) and the mean Ks ranged from 0 (*psbF* and *psbL*) to 0.1978 (*rpl36*). We also calculated the Ka/Ks ratio to evaluate whether selective pressure acted on protein-coding genes. In our study, within the genus *Artemisia*, *accD* evolved under beneficial mutations with a Ka/Ks ratio >1. Three genes (*rps12*, *ycf1*_2, *ndhD*, ranging from 0.5000 to 0.6770) suffered from neutral selection with a Ka/Ks ratio >0.5. However, except for *rps12*, *ycf1*_2, and *ndhD* there were none identified as neutrally evolving between *A*. *selengensis* and other genera, and *ycf1*_1, *rpl33*, *accD*, and *psbH* exhibited neutrally evolution. Upon comparison of Ka/Ks ratios of *Vicia* to other genera species, 14 genes (*atpA*, *ndhB*, *ndhG*, *petB*, *psbC*, *psbD*, *psbH*, *psbZ*, *rpl22*, *rps11*, *rps14*, *rps16*, *rps4*, *ycf4*) were significantly higher (*P* < 0.05), and the difference for *atpF*, *atpB*, *ndhD*, and *rpl14* was highlysignificant at *P* < 0.01 (Fig 5).

### Repeated sequences

In our study, we found 257, 268, 259, 261, 256, 262, 220, 279, and 224 SSRs in *A*. *selengensis*, *A*. *capillaris*, *A*. *frigida*, *A*. *gmelinii*, *A*. *montana*, *C*. *boreale*, *S*. *sessilis*, *D*. *glutinosum*, and *C*. *humilis*, respectively (S5 Table). Among them, the mono-nucleotide was the most common SSR motifs, with 124, 133, 124, 119, 118, 125, 95, 121, 109 in the nine species. Penta-nucleotide and hexa-nucleotide SSRs were limited to only 1–3 for each species. By analyzing the types of SSRs, we found that the AT nucleotide was rich in SSRs. Among them, the content of the A/T mono-nucleotide motifs in *A*. *selengensis*, *A*. *capillaris*, *A*. *frigida*, *A*. *gmelinii*, *A*. *montana*, *C*., *D*. *glutinosum*, and *C*. *humilis* was 98.4%, 98.5%, 99.2%, 98.3%, 98.3%, 98.4%, 100%, 97.5%, and 98.2%, respectively. Furthermore, the content of the AT/TA di-nucleotide motifs in these species were62.5%, 64.6%, 67.4%, 66%, 66%, 63.8%, 63.2%, 60%, and 50%, respectively (S5 Table). The distribution of SSRs among the nine Asteraceae cp genomes showed that 57.4%–62.7% of these SSRs were localized in LSC, whereas 18.6%–21. 9% and 9. 3%–10.2% were localized in SSC and IRa/IRb, respectively. Conversely, the rank order of SSR abundance was intergenic spacers (approximately 45.5%) > protein-coding regions (approximately 40.8%) > intron regions (approximately 10.6%) > rRNA regions (approximately 1.6%) > intergenic spacers and protein-coding regions (approximately 0.9%) > tRNA regions (approximately 0.7%). The *ycf1* gene, which was located in the SSC region was the richest region in *Artemisia* species with 12–20 SSRs. The second richest region was the *ycf2* gene of the IR region with 11–12 SSRs. However, the results for *C*. *boreale*, *S*. *sessilis*, *D*. *glutinosum*, and *C*. *humilis* were different with 15, 21, 18, and 20 SSRs in the *ycf1* gene and 24, 18, 24, and 22 SSRs in the *ycf2* gene, respectively (S5 Table).

In this study, 42, 38, 45, 43, 41, 43, 52, 42, and 38 LDRs were found in *A*. *selengensis*, *A*. *capillaris*, *A*. *frigida*, *A*. *gmelinii*, *A*. *montana*, *C*. *boreale*, *S*. *sessilis*, *D*. *glutinosum*, and *C*. *humilis*, respectively (S6 Table). Most were palindromic repeats and forward repeats. The palindromic repeats accounted for 42.9%, 47.4%, 40.0%, 46.5%, 43.9%, 41.9%, 48.1%, 50%, and 39.5% of the repeats, whereas the positive repeats accounted for 52.4%, 47.4%, 53.3%, 44.2%, 48.8%, 48.8%, 42.3%, 35.7%, and 50%, respectively. In addition, repeats with 30–44 bp lengths were very common in the nine Asteraceae species consisting of 31, 28, 34, 31, 31, 33, 42, 34, and 33, respectively. We also analyzed the distribution of LDRs. Firstly, among these species, 26.3%–39.4% of these repeats were in LSC, whereas 3.5%–34.2%, 19.7%–35.7%, and 19.7%–31.0% were in SSC and Ira/ Irb, respectively. The rich LDRs regions were introns of *ycf3* (LSC), *ycf2*, *rrn4*.*5*-*rnn5* (IR) and an intron of *ndhA* (SSC). Additionally, approximately 38.1% of these repeats were localized in the protein-coding regions, whereas around 39.3% were in the intergenic spacers and approximately 13.8% were in the introns. Specifically, except for *A*. *capillaris*, two dispersed palindromic repeats were found in the *trnS-GGA* gene of the LSC.

### Phylogenetic analysis of *A*. *selengensis*

**Fig 6:**
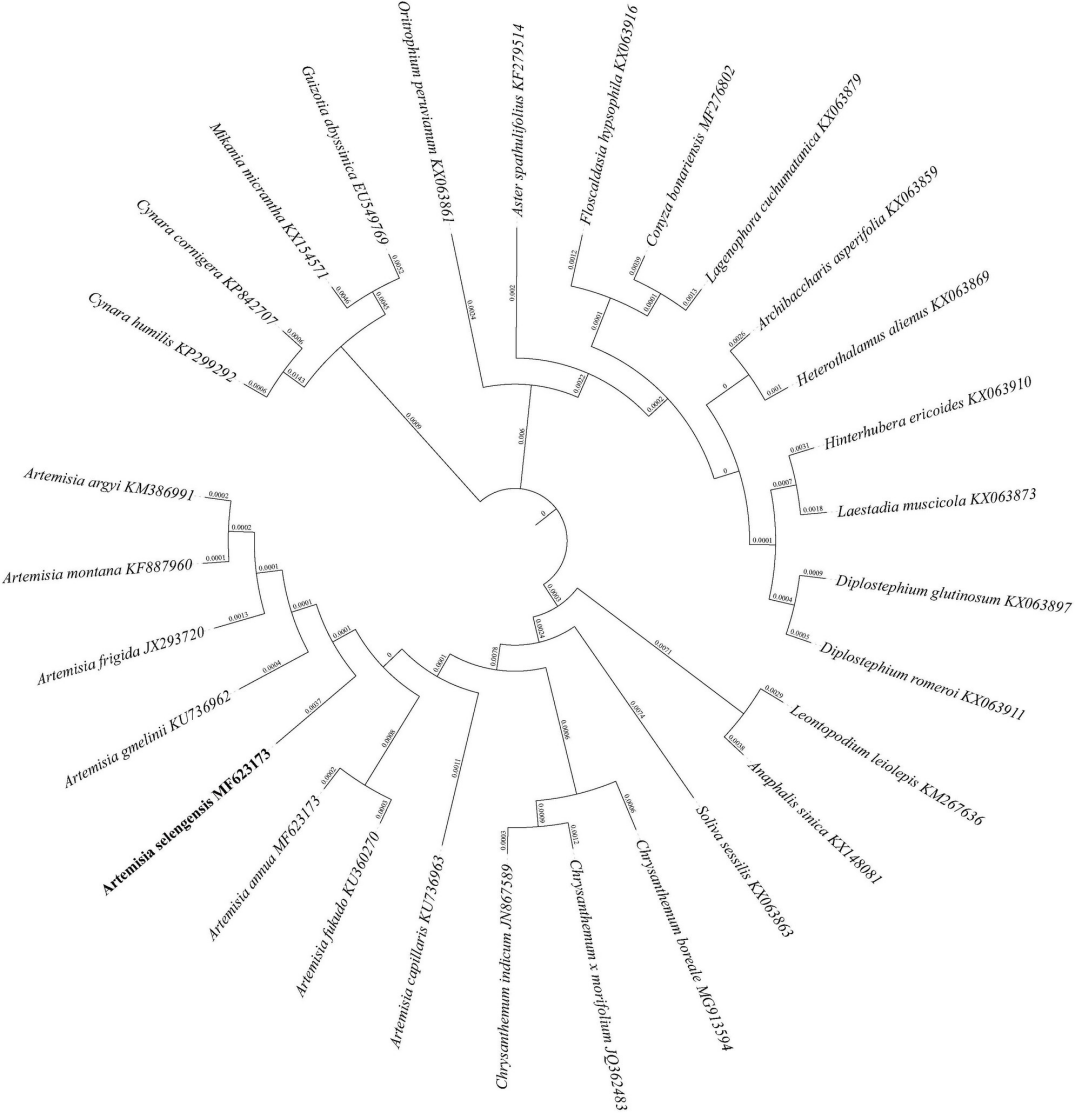
Phylogenetic relationships based on 72 conserved chloroplast protein-coding sequences shared among 29 Asteraceae species with neighbor-joining (NJ) method. *C*. *cornigera* and *C*. *humilis* were selected as the out group.

The NJ phylogenetic tree of five datasets is presented in Fig 6 and S1 Fig. Except for IR and the complete cp genomic tree, *A*. *selengensis*, *A*. *annua*, *A*. *argyi*, *A*. *capillaris*, *A*. *frigida*, *A*. *fukudo*, *A*. *gmelinii*, *A*. *montana*, *C*. *boreale*, *C*. *indicum*, *C*. *x morifolium*, and *S*. *sessilis*, which all belong to the tribe Anthemideae, were located in the same clade. By analyzing the LSC, SSC, and 72 shared-protein-sequences tree, *S*. *sessilis* was the well-supported basal taxon, but the relationship between *Artemisia* and *Chrysanthemum* was different. *A*. *annua* and *A*. *frigida* formed a new branch, which was a sister group with another branch constituted by the remaining six *Artemisia* species in the LSC and 72 shared-protein-sequences tree. However, this new branch contained five species in the SSC tree: *C*. *boreale*, *C*. *indicum*, *C*. *x morifolium*, *A*. *annua*, and *A*. *frigida*. The evolutionary distances were also calculated. The results showed that the closest species to *A*. *selengensis* was *A*. *capillaris* (0.0017), *A*. *argyi* (0.0027), *A*. *gmelinii* (0.0040), *A*. *montana*, and *A*. *fukudo* (0.0006), and *A*. *gmelinii* and *A*. *montana* (0.0042) in the complete cp genome, LSC, SSC, IR, and 72 shared-protein-sequences trees, respectively.

## Discussion and conclusion

The genomic length of chloroplast within the *Artemisia* genus was similar, ranging from 151,056 bp (*A*. *capillaris*) to 151,318 bp (*A*. *gmelinii*) with only a 255 bp difference. Moreover, the available cp genomes of *Artemisia* had conserved genomic organization, GC contents, and gene order (Table 3). Like most angiosperms, *A*. *selengensis* had a typical quadripartite structure and is an AT-rich species. The GC content of *A*. *selengensis* (37.46%) was quite similar to that of other Asteraceae species, such as *A*. *capillaris* (37.46%) [25], *A*. *frigida* (37.48%) [23], *A*. annua (37.48%) [24], and *S*. *sessilis* (37.46%) [41] belonging to the order Asteroideae, and other species in the order Carduoideae, such as *C*. *humilis* (37.70%) and *C*. *cornigera* (37.71%) [56].

However, when we compared *A*. *selengensis* with other genera in the Asteraceae family, some differences which may indicate evolutionary events were found. Normally, the SSC region of most Asteraceae species has been inverted relative to the *Nicotiana tabacum* chloroplast genome, which is often regarded to be unaltered [57]. However, in our study, we noticed that the SSC region of five species within the *Artemisia* genus had no rearrangement but was inverted in comparison with other genera in the Asteraceae family. This event is in agreement with a previous study on *A*. *frigida*, which has been called “re-inversion” [23]. Actually, except for *Artemisia* species, this re-inversion event was also found in *Carthamus tinctorius* (KP404628) [58], *Centaurea diffusa* (NC024286), and one reported *Lactuca sativa* (NC007578) [59]. One possible explanation for these results may be that the SSR region is an inversion “hotspot” and the re-inversion event can be noticed in closely related individuals. However, even in individual plants, there will be SSC re-inversion events as well. For example, the SSC regions of two cp genome sequences of *Lactuca sativa* (NC007578 and DQ383816) presented different orientations [60, 61]. Although some hypotheses have been proposed for the mechanism of different SSC orientations within and among individuals, including intramolecular recombination between the two IR regions [60] and recombination-dependent DNA replication of the cp genome [62], the regulation mechanism of the presence of the re-inversion event within and among individuals is still unclear.

The border between four junctions usually differs among plants [63]. Detailed comparisons of IR boundaries of intro-generic and inter-generic species in the Asteraceae family suggested that wide ranges of expansions and contractions of IR are very common evolutionary events. As a result, the pseudogenes, *ycf1* and *rps19*, were present at the IRa/SSC and IRb/LSC boundaries, respectively. We also identified an unequal duplicate phenomenon of the *ycf1* gene and overlapped regions between *ycf1* and *ndhF*. Actually, the sizes of IRs can change from 10 kb (in liverworts) to 76 kb (in Pelargonium) in land plants [64, 65]. Most angiosperms have a 20–25 kb IRs. Wang et al.(2008) proposed three types to explain the expansion and contraction of IR/LSC junctions in angiosperms. Type I relates to intact *trnH* and *rps19* genes being seated in IRa and IRb, respectively, and *rps19* is seated downstream of *trnH*. In Type II there is a partial *rps19* in Ira, which is situated between *rpl2* and *trnH*. This type coincides with our study and has been found in some eudicots. Type III relates to the same *trnH*-*rps19* cluster in IRa or IRb. Several mechanisms have been proposed to explain why successive IR expansions can lead to floating of the four junctions, such as homologous dispersed repeat recombination in *Geranium* [66].

Except for the large-scale evolutionary events in the cp genome of *A*. *selengensis*, we also identified the most variable regions by calculating the pairwise distances of IGSs, introns, and protein-coding sequences of nine Asteraceae species relative to *A*. *selengensis*. K2p values are an effective method for estimating evolutionary rates of nucleotide sequences [46]. In our study, the *ndhD*_*psaC* (116 bp), *psaJ*_*rpl33* (439 bp), *trnH-GUG*_*psbA* (382 bp), *rps18*_*rpl20* (264 bp), *ccsA*_*ndhD* (200 bp), and *rpl32*_*trnL-UAG* (880 bp), which presented higher K2p values, indicated that these regions exhibited accelerated mutation rates within the *Artemisia* genus. The Ka/Ks ratio is used to evaluate whether selective pressure acts on protein-coding genes and is an important indicator for studying gene evolution. When Ka/Ks > 1 (= 1; <1), the gene was subjected to positive selection (neutral selection; purifying selection) [46]. In our study, *accD* evolved under beneficial mutations with a Ka/Ks ratio >1. Three genes (*rps12*, *ycf1*_2, *ndhD*, ranging from 0.5000 to 0.6770) suffered from neutral selection with a Ka/Ks ratio > 0.5.

Repeats play an important role in various rearrangements, such as additions, deletions, or large inversions [47]. Therefore, we analyzed SSRs and LDRs in cp genomes of the nine Asteraceae species and found 220–279 SSRs and 38–52 LDRs in each individual. Mono-nucleotide, palindromic, and forward repeats were the most common repeated sequences. Nine Asteraceae cp genomes presented a highly similar pattern of SSRs or LDRs distribution. Firstly, more than half of the SSRs was present in the LSC region, and approximately 45.5% and 40.8% of SSRs were in IGSs and protein-coding regions, respectively. Secondly, approximately 30% of LDRs were localized in the LSC, IRa, or IRb regions, approximately 39% of LDRs were in IGSs or the protein-coding regions. The same situation is also found in other species, such as Fabaceae [47] and Sapindaceae species. Then, we associated repeat distribution with different regions and found that *ycf2*, *ycf1*, *ycf3*, *rrn4*.*5 and rrn5* were the richest regions (n > 10). In a word, these SSCs and LDRs present in our study represent important genetic maker resources that can be used to expand research on *Artemisia* species.

Five datasets, including the complete cp genomes, LSC, IR, SSC DNA sequences, and 72 shared protein sequences, reconstructed the *Artemisia* and *Asteraceae* phylogenetic relationship. However, different datasets produced different topological structures (Fig 6 and S1 Fig). Among them, LSC and the 72 shared-protein-sequences tree showed the most similar topological structures and were consistent with the phylogeny of 21 Korean *Artemisia* species reconstructed by *trnL*_*trnF* markers [27]. However, although some *Artemisia* cp data have been published, other studies contained only one to four *Artemisia* species [22–26], and it is difficult to obtain more phylogenetic data to support our results.

In summary, a new cp genomic resource *A*. *selengensis* was presented. This study filled the gap in *A*. *selengensis* genomic resources, and provides novel insights into evolutionary dynamics in an important medicinal resource clade: *Artemisia*. Our results revealed that the available cp genomes of *Artemisia* were well conserved in terms of genomic length, GC contents, gene organization, and order. Furthermore, some differences, which may indicate evolutionary events, were found. Firstly, a re-inversion event of the SSC region within the *Artemisia* genus was identified, but the regulation mechanism of the presence of the re-inversion event within and among individuals is still unclear. Secondly, the pseudogenes *ycf1* and *rps19*, an unequal duplicate phenomenon of the *ycf1* gene, and overlapping regions between *ycf1* and *ndhF* were identified at the IR/SSC or IR/LSC boundaries because of the expansion and contraction of the IR region. Last but not least, the highly variable regions (*ndhD*_*psaC*, *psaJ*_*rpl33*, *trnH-GUG*_*psbA*, *rps18*_*rpl20*, *ccsA*_*ndhD*, *rpl32*_*trnL-UAG*, *accD*, *rps12*, *ycf1*_2 and *ndhD*) within *Artemisia*, which indicated fast-evolving events, were found. The analysis of repeated sequencesshowed that Asteraceae cp genomes presented a highly similar pattern of SSRs or LDRs distribution. The phylogenetic analysis of five datasets showed that LSC and 72 shared-protein-sequences may be more useful in the reconstructed *Artemisia* and *Asteraceae* phylogenetic relationship. This study will be useful for further studies to illuminate the genetic diversity and evolutionary history of Asteraceae.

## Supporting information

S1 Table K2p values of introns and intergenic spacers of nine Asteraceae species relative to *A*. *selengensis* (AS). (XLSX)

S2 Table Non-synonymous mutatation rate of protein-coding sequences of nine Asteraceae species relative to *A*. *selengensis* (AS). (XLSX)

S3 Table Synonymous mutatation rate of protein-coding sequences of nine Asteraceae species relative to *A*. *selengensis* (AS). (XLSX)

S4 Table Ka/Ks ratio of protein-coding sequences of nine Asteraceae species relative to A. selengensis (AS). (XLSX)

S5 Table Simple sequence repeats in the nine Asteraceae chloroplast genomes investigated in this study. (XLSX)

S6 Table Longer dispersed repeats in the nine Asteraceae chloroplast genomes investigated in this study. (XLSX)

S1 Fig Phylogenetic relationships based on whole chloroplast genomes (A), LSC region (B), SSC region (C), and IR region (D) 72 among 29 Asteraceae species with neighbor-joining (NJ) method.
(TIF)
