# Supplementary figures and images for: Detecting useful genetic markers and reconstructing the phylogeny of an important medicinal resource plant, Artemisia selengensis, based on chloroplast genomics
Source: PLoS One. 2019 Feb 4;14(2):e0211340. doi: 10.1371/journal.pone.0211340 (PMC6361438; doi:10.1371/journal.pone.0211340)

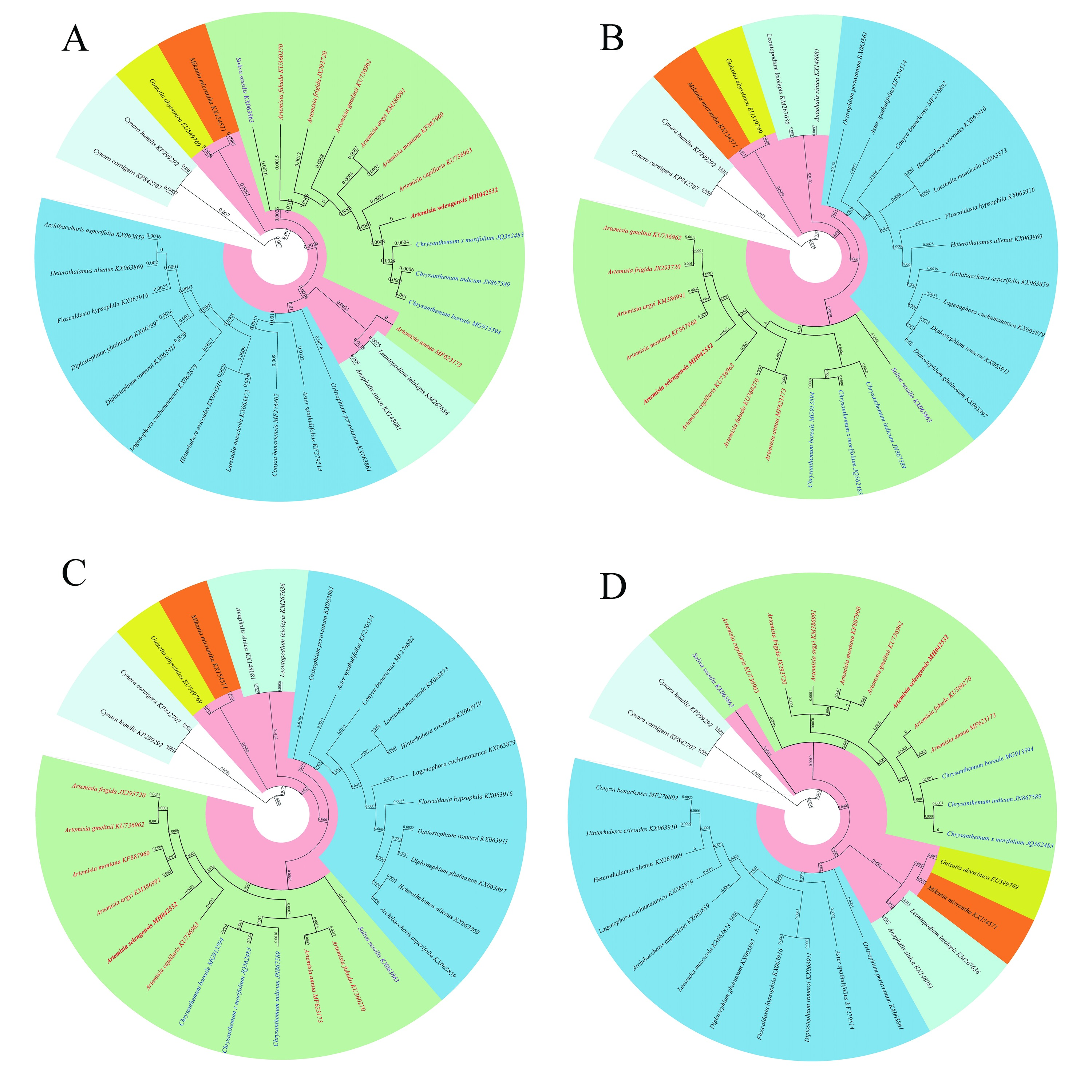

Supplement: S1 Fig — Phylogenetic relationships based on whole chloroplast genomes (A), LSC region (B), SSC region (C), and IR region (D) 72 among 29 Asteraceae species with neighbor-joining (NJ) method. (TIF) [file pone.0211340.s007.tif]
